# Feasibility of image-guided radiotherapy based on helical tomotherapy to reduce contralateral parotid dose in head and neck cancer

**DOI:** 10.1186/1471-2407-12-175

**Published:** 2012-05-11

**Authors:** Nam P Nguyen, Paul Vos, Vincent Vinh-Hung, Misty Ceizyk, Lexie Smith-Raymond, Michelle Stevie, Benjamin Slane, Alexander Chi, Anand Desai, Shane P Krafft, Siyoung Jang, Russ Hamilton, Ulf Karlsson, Dave Abraham

**Affiliations:** 1 Department of Radiation Oncology, University of Arizona, Tucson, AZ, USA; 2 Biostatistics, East Carolina University, Greenville, NC, USA; 3 Department of Radiation Oncology, University Hospitals of Geneva, Geneva, Switzerland; 4 Department of Radiation Oncology, University of West Virginia, Morgantown, VA, USA; 5 Department of Radiation Oncology, University of Pittsburg, Pittsburg, PA, USA; 6 Department of Radiation Oncology, Marshfield Clinic, Marshfield, WI, USA; 7 University of Arizona, 1501 N. Campbell Ave., Tucson, AZ 85724-5081, USA

**Keywords:** Head and neck cancer, Tomotherapy, Parotid sparing

## Abstract

**Background:**

To evaluate the feasibility of image-guided radiotherapy based on helical Tomotherapy to spare the contralateral parotid gland in head and neck cancer patients with unilateral or no neck node metastases.

**Methods:**

A retrospective review of 52 patients undergoing radiotherapy for head and neck cancers with image guidance based on daily megavoltage CT imaging with helical tomotherapy was performed.

**Results:**

Mean contralateral parotid dose and the volume of the contralateral parotid receiving 40 Gy or more were compared between radiotherapy plans with significant constraint (SC) of less than 20 Gy on parotid dose **(**23 patients) and the conventional constraint (CC) of 26 Gy (29 patients). All patients had PTV coverage of at least 95% to the contralateral elective neck nodes. Mean contralateral parotid dose was, respectively, 14.1 Gy and 24.7 Gy for the SC and CC plans (*p* < 0.0001). The volume of contralateral parotid receiving 40 Gy or more was respectively 5.3% and 18.2% (*p* < 0.0001)

**Conclusion:**

Tomotherapy for head and neck cancer minimized radiotherapy dose to the contralateral parotid gland in patients undergoing elective node irradiation without sacrificing target coverage.

## Background

Xerostomia remains one of the most common sequelae of head and neck cancer radiotherapy because of excessive radiation to the parotid glands [1-3]. Radiotherapy induces apoptosis of the acinar glands leading to decreased salivary production and decreases patient’s quality of life [4,5]. The severity of dry mouth is proportional to the radiation dose delivered to the parotid glands [6]. Conventional radiotherapy with two lateral fields leads to significant saliva reduction because of its inability to spare the parotid glands from the radiation fields. Introduction of intensity-modulated radiotherapy (IMRT) leads to significant sparing of the parotid glands in patients with clinically negative neck nodes because of rapid dose fall of from the target volume [7,8]. Mean dose to the parotid gland is usually kept at 26 Gy or less because of the observed recovery of saliva production from the parotid gland with mean radiation doses up to 26 Gy [9]. However, in patients undergoing concurrent chemoradiation, damage to the parotid gland may occur with a lower dose because of the radiosensization effect of chemotherapy [10]. Thus, minimizing radiation dose to the parotid gland remains one of the challenges for radiotherapy of head and neck cancer. Avoidance of contralateral neck irradiation is one of the most effective means of sparing the contralateral parotid gland from radiation [11]. However, in patients with large tumors (T3-T4), clinically positive neck nodes, or tumors affecting an anatomic location with rich lymphatic drainage, elective nodal irradiation (ENI) of the contralateral neck is essential in optimizing regional control in the contralateral neck [12]. In patients with unilateral cervical lymph node metastases, the ipsilateral parotid gland is not spared from radiation because of the risk of under-dosing level II lymph node and to prevent recurrence in the peri-parotid lymph node [13,14]. By sacrificing the ipsilateral parotid gland and purposely under-dosing the contralateral level II lymph nodes, significant sparing of the contralateral parotid gland may be achieved with adequate local control [14]. Ideally a radiotherapy technique that can achieve adequate coverage of the regional lymph nodes at risk while minimizing contralateral parotid gland irradiation will lead to significant improvement in patients’ quality of life as well as effective regional control in the neck. Image-guided radiotherapy (IGRT) has been recently introduced to decrease normal tissue toxicity because of the rapid dose fall off compared to IMRT [15]. Helical Tomotherapy is an image-guided radiotherapy technique incorporating daily megavolt (MV) computed tomography (CT) planning and dynamic rotational IMRT [16]. Effectiveness of Tomotherapy-based IGRT has been proven to be superior to conventional IMRT for larynx and pharyngeal muscles sparing in patients with non-laryngeal and -hypopharyngeal cancers even in the presence of neck node metastases [17]. We investigate in this report the feasibility of Tomotherapy to reduce parotid gland irradiation in head and neck cancer patients undergoing elective neck node irradiation.

## Methods

The medical records of 52 patients undergoing radiotherapy for head and neck cancer at the University of Arizona Radiation Oncology Department from December 2007 to March 2010 were retrospectively reviewed following institutional review board (IRB) approval. Patients with ipsilateral or bilateral clinical N0 neck receiving bilateral neck irradiation were selected for this study. Patients who underwent unilateral neck irradiation or presented with bilateral neck nodes metastases at diagnosis were excluded because of the high recurrence risk associated with under-dosing level II and periparotid lymph nodes [13,14] . All patients were treated with the whole field (WF) IGRT technique on a helical Tomotherapy **(Hi-Art Tomotherapy**^**R**^**)** unit by a single radiation oncologist with the same radiotherapy technique. Prior to treatment, each patient was simulated in the supine position with a head and neck aquaplast mask for treatment immobilization. A computed tomography (CT) scan with and without intravenous (IV) contrast for treatment planning was performed in the treatment position. The head and neck areas from the vertex to the mid thorax were scanned with a slice thickness of 3 mm. CT scan with IV contrast was employed to outline the tumor and grossly enlarged cervical lymph nodes for target volume delineation. Radiotherapy planning was performed on the non-contrast CT scan to avoid possible interference of contrast density on calculations radiotherapy isodose distributions. Diagnostic positron emission tomography (PET)-CT scan planning for tumor imaging was also incorporated with CT planning for target volume delineation whenever available. A 0.5 cm bolus material was placed on any area of the skin involved by the tumor and on any palpable cervical lymph nodes. Normal organs at risk for complication were outlined for treatment planning (spinal cord, brain stem, bilateral cochlea, mandible, parotid glands, larynx, pharyngeal muscles, bilateral eyes, and oral cavity). Radiation therapy dose was similar for all patients with the integrated boost technique to decrease treatment toxicity.

The tumor and grossly enlarged lymph nodes (GTV1) on CT scan with a margin of 5 mm to 1 cm depending on anatomic location (PTV1) were treated to 70 Gy in 35 fractions (2 Gy/fraction). The area at high risk-PTV2 (at least 1 cm around PTV1 if PTV1 was located in the soft tissues with no anatomic barriers such as fatty tissues or muscles) was treated to 63 Gy in 35 fractions (1.8 Gy/fraction). If PTV1 was located adjacent to a bony structure which constituted a natural barrier to tumor spread and if there was no bony invasion observed on CT scan, no additional dose level will be added. In that specific case, PTV1 became PTV2. We added PTV2 because of our earlier experience with the IMRT technique when the target volume could not be visualized to avoid marginal miss. The low risk -PTV3 or elective nodal irradiation (subclinical regional lymph nodes with 5 mm margins) for tumor spread was treated 56 Gy in 35 fractions (1.6 Gy/fraction).

Minimal targetcoverage was 95% of the prescribed dose for all targets with at least 99% of the prescribed dose delivered to gross tumor and involved cervical lymph nodes. The lymph nodes in the ipsilateral neck including the retropharyngeal lymph nodes were treated to the base of skull if there was any cervical lymph node enlargement (or PET-positive lymph nodes). Contralateral uninvolved lymph nodes were treated prophylactically with the C1 vertebrae as the superior border of the treatment field. Mean dose to the contralateral parotid was kept below 26 Gy (conventional constraint) if there was no cervical lymph node enlargement in the historical control group (29 patients). However, if this parotid dose was not achievable, the lowest mean parotid dose that allowed target coverage was accepted. Mean dose to contralateral parotid was kept below 20 Gy (significant constraint) for the study group (23 patients) consistent with the quantitative analyses of normal tissue effects in clinic recommendation (QUANTEC) [18]. These 23 patients were treated after publication of the QUANTEC recommendations. In patients with unilateral neck nodes, there was no constraint on the ipsilateral parotid to avoid any marginal miss because of the close proximity of the parotid gland to the jugular digastric lymph node. There was also no attempt to spare the submandibular glands from radiation. Dose constraints for other normal organs at risk (OAR) for complications were: spinal cord (45 Gy), brain stem (50 Gy), optic chiasm (45 Gy), mandible (70 Gy to less than 30% of the mandible). Doses to larynx and pharyngeal muscles for non-laryngeal and - hypopharyngeal cancers were kept between 20–40 Gy if feasible. Statistical analysis was performed with the Welch’s *t* test. A difference of <0.05 was considered statistically significant.

We identified 52 patients with invasive squamous cell carcinoma of the head and neck treated at the University of Arizona Radiation Oncology departmentfrom 2007 to 2010. Median age at diagnosis was 63 years-old (range: 25–92 years old). There were 49 males and 3 females. Disease sites were respectively: oropharynx (15), oral cavity (15), larynx (10), hypopharynx (4), parotid (4), unknown (3), and nasopharynx (1). There were two stage I, three stage II, 21 stage III, 16 stage IVA, 7 stage IVB, two stage IVC, and one recurrence. 41 patients had unilateral neck metastases and 11 patients had no neck nodes. Treatment consisted of: radiotherapy alone (10), and concurrent chemoradiation (42). Table 1 summarizes the patient characteristics. Table 2 summarizes patient clinical outcome in both groups. Two patients in the study group died during treatment from aspiration pneumonia (1) and disease progression (1). No patient in either groups had recurrences in the parotid area. As this is a retrospective study, there was no clinical information on the impact of parotid sparing on the severity of xerostomia.

**Table 1:** Patient characteristics

		**Study**	**Historical control**	**Total**
Patient No		23	29	52
Age				
	Median	66	60	63
	Range	25–92	26–90	25–92
Sex				
	Male	21	28	49
	Female	2	1	3
	Squamous histology	22	30	52
Tumor sites				
	Oropharynx	5	10	15
	Oral cavity	7	8	15
	Parotid (primary or metastates)	3	1	4
	Larynx	4	6	10
	Hypopharynx	2	2	4
	Unknown	1	2	3
	Nasopharynx	1	0	1
Stages				
	I	0	2	2
	II	2	1	3
	III	9	12	21
	IVA	6	10	16
	IVB	3	4	7
	IVC	2	0	2
	Recurrence	1	0	1
T stages				
	Tx	1	2	3
	T1	1	4	5
	T2	13	10	23
	T3	3	8	11
	T4	4	5	9
	Recurrence	1	0	1
Neck nodes				
	N0	3	8	11
	N1	13	9	22
	N2	4	10	14
	N3	2	2	4
	Recurrence	1	0	1
Treatement				
	Radiotherapy alone	5	5	10
	Chemoradiation	18	24	42

Study: Mean parotid dose constraints less than 20 Gray; Control: Mean parotid dose constraints 26 Gy.

**Table 2:** Clinical outcome following image-guided radiotherapy of the control group and the study group

	**Study (23)**	**Historical control (29)**
Lost for follow-up	1	0
Follow-up (months)		
Median	20	31
Range	1–39	1–48
Alive	14 (63.6%)	21 (72.4%)
Local recurrences	5 (22.7%)	5 (17.2%)
Regional recurrences	2 (9%)	2 (6.9%)
Distant metastases	2 (9%)	4 (13.8%)
Death during treatment	2 (1 aspiration pneumonia, 1 disease progression)	0

## Results

**Figure 1:**
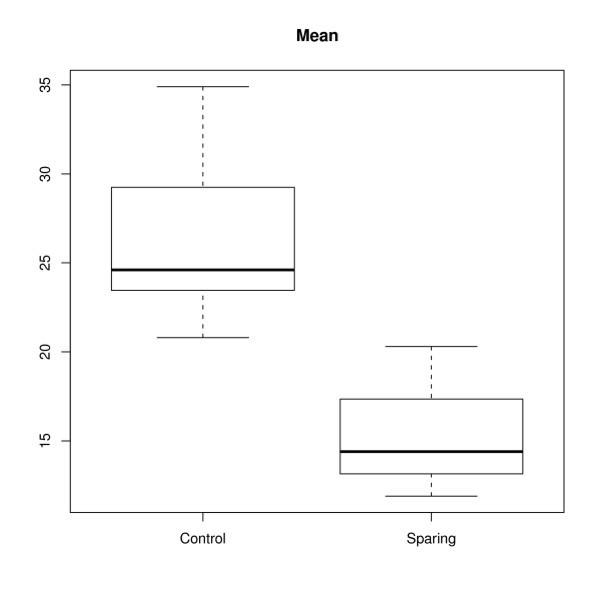
Mean contralateral parotid dose in head and neck cancer patients undergoing image-guided radiotherapy with conventional (historical control group) and significant constraint (study group) on parotid dose

**Figure 2:**
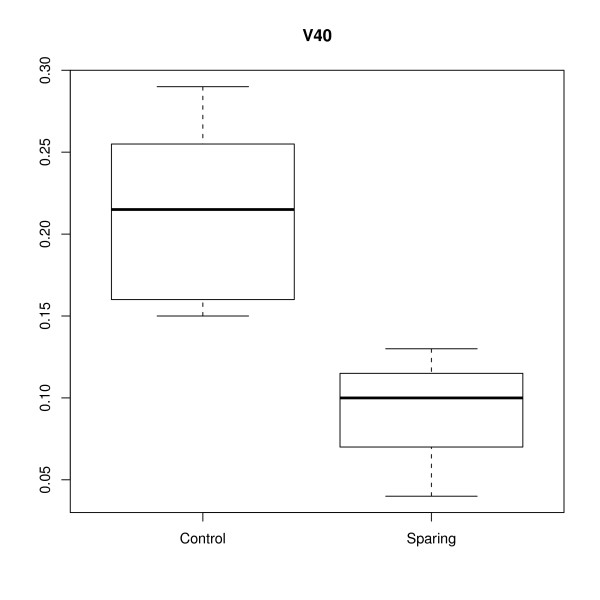
Volume of contralateral parotid gland receiving 40 Gy in head and neck cancer patients undergoing image-guided radiotherapy with conventional (historical control group) and significant constraint (study group) on parotid dose

**Figure 3:**
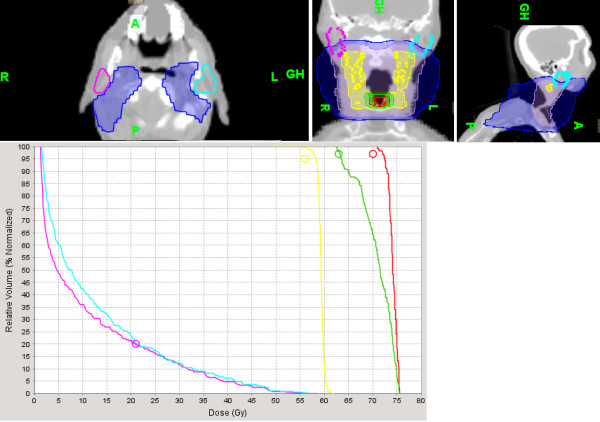
**Illustration of the effectiveness of Tomotherapy to decrease radiation dose to both parotids in a patient with locally advanced laryngeal cancer and negative neck nodes on PET-CT scan (T3N0M0).** The tumor bed and bilateral levels II-IV neck nodes were treated to 70 Gy (red) and 56 Gy (yellow) respectively. Despite the close proximity of the parotid glands to the 56 Gy isodose line, mean parotid dose was 11.2 Gy for the right and 12.7 Gy for the left

Mean contralateral parotid dose was, respectively 14.1 Gy (range: 6.5 Gy to 19.9 Gy) and 24.7 Gy (range: 20.1 Gy to 34 Gy) for the study group (SC) and historical control group (CC) [95% CI: 8.5 to 12.5] (*p* < 0.0001). The volume of the contralateral parotid receiving 40 Gy was respectively, 5.3% (range: 0 to 14%) and 18.2% (range: 3% to 35%) [95% CI: 9.5 to 16.1] (*p* < 0.0001). Figures 1 and 2 illustrate respectively the mean dose and volume of the contralateral parotid gland receiving 40 Gy. There was no significant difference in PTV coverage between the two groups. Mean PTV coverage for the historical control group and study group was respectively 96.1% (range: 95% to 98%) and 96.8% (range: 95% to 98%). Figure 3 illustrates the effectiveness of Tomotherapy to decrease radiation dose to both parotids in a patient with locally advanced laryngeal cancer and negative neck nodes. We also assessed the mean bilateral parotid dose in both groups even though we did not put any constraint on the ipsilateral parotid dose. We would like to verify that Tomotherapy can selectively spare the contralateral parotid gland because of the beams optimization without excessively increasing dose to the ipsilateral parotid gland. For this analysis, patients with primary tumors located in the parotid gland were excluded because of the high dose to the tumor (70 Gy). Mean bilateral parotid dose (right and left) was respectively 28.2 Gy and 30.8 Gy for the study group and control group [95% CI: -1.7 to 7] (*p* = 0.2). We also investigated whether the parotid sparing efficacy of Tomotherapy was not due to excessive dose to the surrounding normal structures such as the spinal cord and mandible.

We compared the maximum spinal cord and mean mandibular dose between patients with oropharyngeal, oral cavity, and laryngeal-hypopharyngeal cancer in the study group and historical control group. Tumors of the larynx and hypopharynx were grouped together because of the small number of patients and their close anatomic location. There were no significant difference in spinal cord or mandibular dose. Table 3 summarizes spinal cord and mandibular dose of the study and historical control group**.** We also raised the question whether Tomotherapy can effectively spare both parotid glands in the absence of cervical lymph node. Eleven patients did not have cervical lymph nodes involvement, three in the study group and eight in the control group. Mean total parotid dose was respectively 15.5 Gy and 26.2 Gy for the study group and the control group [95% CI: 2.3 to 19.1] (*p* = 0.02)

**Table 3:** Maximum spinal cord dose and mean mandibular dose (Gray) in the study group and historical control group for oropharyngeal, oral cavity, and laryngeal-hypopharyngeal cancers

**Site**	**Spinal cord**			**Mandibular**		
	Study	Control	p value	Study	Control	p value
Oropharynx	35.2	39.3	0.1	39.2	44.9	0.4
Oral cavity	33.6	36.9	0.1	55.4	57.2	0.4
Larynx and hypopharynx	34.2	36.1	0.5	30	31.8	0.8

## Discussion

To our knowledge, the present work represents the first study attempting to reduce the dose to the parotid in patients undergoing elective neck node radiotherapy for head and neck cancer employing helical tomotherapy with IGRT. All patients in our study were treated with Tomotherapy. Tomotherapy provides significant dose reduction to the parotid gland without compromise of target volume dose. In an effort to reduce parotid dose, our main concern was to treat the regional neck nodes at risk for subclinical disease effectively to 56 Gy in 35 fractions. We discovered in a previous study that Tomotherapy-based IGRT has been effective in decreasing laryngeal and pharyngeal dose compared to conventional IMRT in patients with head and neck cancer [17]. Thus, our initial goal was to achieve a modest reduction of mean parotid dose below 20 Gy with Tomotherapy while covering the elective neck PTV with at least 95% of the prescribed dose. Our parotid dose reduction was based on a review of the literature of normal tissue toxicity following head and neck radiotherapy: QUANTEC recommends a mean parotid dose of 20 Gy or less if only one parotid gland can be spared from radiotherapy [18]. As we acquired more experience, we gradually developed a technique that provided significant reduction of parotid dose regardless of the cancer anatomic site. In selected patients, a mean parotid dose of 10 Gy or less may be achieved. This is particularly important in patients who underwent concurrent chemoradiation for oral cavity and oropharyngeal tumors with unilateral lymph nodes metastases when the ipsilateral parotid, submandibular, and minor salivary glands received high radiation dose. These patients remained at high risk for severe xerostomia following head and neck radiotherapy because of the tumor location and disease extent. Preserving one parotid gland from excessive radiotherapy may improve patient quality of life without compromising local control.

Many studies have looked into relationships between parotid radiation dose and degree of xerostomia severity. Additionally, other studies have correlated the mean parotid dose with the subsequent reduction of salivary flow. Therefore, we have reviewed studies in which dose-volume histograms were performed with treatment planning head and neck CT scan reporting the mean parotid dose and the associated risk of xerostomia. Eisbruch et al. [9] reported that maintaining mean parotid dose below 26 Gy lead to recovery of stimulated salivary flow at one year following head and neck radiotherapy. Munter et al. [19] also corroborated that reducing mean parotid dose below 26 Gy with IMRT significantly preserved salivary function. Chao et al. [20] reported a direct correlation between the mean parotid dose and the decreased rate of stimulated salivary flow estimated to be 4% per Gy of mean parotid dose. However, other studies have suggested alternated parameters of parotid dose levels for the development of xerostomia following head and neck radiotherapy. Bussels et al. [3] reported a 50% loss of salivary function with mean parotid dose of 22.5 Gy at 7 months following head and neck cancer IMRT. Roesink et al. [6] reported increased risk of developing severe xerostomia with mean parotid dose of 39 Gy. Saarilahti et al. [21] reported minimal reduction of stimulated salivary flow with mean parotid dose of 18 Gy or less and marked decreased of saliva production with parotid dose between 20–30 Gy. Table 4 summarizes mean parotid dose reported in the literature and radiotherapy effect on salivary gland production.

**Table 4:** Mean parotid dose in relation to reduction of salivary flow in studies with dose-volume histogram based on planning head and neck computed tomography scans

**Study**	**Patient No**	**Technique to assess salivary flow**	**Mean parotid dose**	**Evaluation time**
Teshima et al. [1]	20	Saxon test	30 Gy	NS
Bussels et al. [3]	16	Scintigraphy	22.5 Gy	7 months
Roesink et al. [6]	108	Suction cup	39 Gy	months
Eisbruch et al. [9]	88	Suction cup	26 Gy	12 months
Cerezo et al. [11]	16	Spitting cup	4.7 Gy (contralateral unirradiated parotid)	12 months
Munter et al. [19]	18	Scintigraphy	26 Gy	6 months
Chao et al. [20]	41	Spitting cup	30 Gy	6 months
Saarilahti et al. [21]	17	Spitting cup	25.5 Gy	12 months

Gy: Gray; NS: not specified.

The discrepancies between these studies may be due to many factors such as parotid size, heterogeneity of IMRT dose distribution within the parotid glands, inclusion of patients treated with chemoradiation which increased radiosensibility of the salivary glands, and inter-patient variability in loss of salivary function. In patients who underwent ipsilateral neck irradiation for well lateralized oral cavity or oropharyngeal tumors, the mean dose to the contralateral parotid gland was 4.7 Gy [11]. The low contralateral parotid dose decreased xerostomia severity and improved patient quality of life. Ortholan et al. [22] also corroborated that reduction of contralateral parotid gland dose lead to recovery of salivary function. If the volume of the contralateral parotid gland receiving 40 Gy (V40) was kept below 33%, complete salivary production recovered after two years. It was postulated that sparing of the contralateral parotid gland allowed it to compensate for the low salivary production of the ipsilateral gland. The feasibility of this approach has been demonstrated in improving patient quality of life [23]. Thus, because a large proportion of the study population received concurrent chemotherapy for locally advanced head and neck cancer which had an additive effect on salivary flow, we devised a new policy to preserve salivary gland function by reducing mean contralateral parotid dose with Tomotherapy.

Helical Tomotherapy has been proven to deliver a sharper dose gradient compared to conventional IMRT, thus reducing radiation dose to the parotids without compromising target coverage [13,24]. Mean and V40 contralateral parotid dose was respectively 14.1 Gy and 24.7 Gy and 5.5% and 18.2% for the study and historical control populations (*p* < 0.0001). Even though we lack information on salivary flow following radiotherapy with parotid sparing Tomotherapy, our study demonstrates the feasibility of this new technique to potentially improve patient quality of life because of low radiation dose to the contralateral parotid gland. In a pilot study, Voordeckers et al. [25] reported the feasibility of Tomotherapy to conserve salivary function if 46% of the unilateral parotid volume received a dose less than 60 Gy. Maes et al. [26] demonstrated that salivary gland function may be preserved if the contralateral parotid gland received 20 Gy or less. Using scintigraphy to measure salivary flow following radiotherapy with 3-dimensional (3-D) conformal radiotherapy, 70% of the parotid function may recover six months following treatment. Thus, as the study mean parotid dose was 14.1 Gy and only a minimal volume of the parotid gland received more than 40 Gy (5.5%), we can expect that our patients may benefit from significant xerostomia reduction. We emphasize that parotid dose reduction does not compromise target coverage as there was no difference in PTV coverage between the two groups. In addition, daily megavoltage computed tomography (MVCT) imaging for patient set up allows for on-line correction of patient positioning variation and insures accurate dose delivery and sparing of the parotid glands [27]. We demonstrate that reduction of contralateral parotid dose was achieved without any increase of radiation dose to the ipsilateral parotid gland, spinal cord, or mandible. In patients with no cervical lymph nodes metastases, mean parotid dose in the study group was significantly lower than the historical control group. Even though the patient number with N0 node was small, we postulate that Tomotherapy provides optimal sparing of the parotid gland while preserving target coverage because of the high number of beamlets associated with dynamic rotational IMRT and sharp dose gradient [16]. Our clinical study also confirmed the dosimetric experience of other investigators about the potential of Tomotherapy for improving target coverage while minimizing radiation dose to the normal tissues in head and neck cancer patients. Lee et al. [28] compared the dose volume histogram (DVH), conformity index (CI), homogeneity index (HGI) and minimal dose to 1 cc (Dmin-1 cc) of 20 nasopharyngeal cancer patients treated with Tomotherapy and re-planned with step-and-shoot IMRT. Tomotherapy significantly improved CI and HI of the PTV while significantly reduced radiation dose to the other organs at risk for complications. Mean dose to the parotid glands was 28% less compared to IMRT. Jacob et al. [29] also corroborated the superiority of Tomotherapy in providing better coverage to the target volume (higher minimum dose) compared to RapidArc and dynamic IMRT. Parotid dose was lowest with Tomotherapy. The improved therapeutic ratio of Tomotherapy was also reported in two other dosimetric studies [30,31], thus arguing that Tomotherapy may be best suited to reduce xerostomia compared to other IMRT techniques if significant constraint was placed on parotid dose. The limitations of the present study include the retrospective nature of the study, the small number of patients, and the lack of information on salivary production before and after radiotherapy. We also did not have information on the impact of parotid sparing on the severity of xerostomia and patient quality of life. Nevertherless, we hope that our study will encourage other institutions to investigate the potential of Tomotherapy to spare the parotid gland from possible excessive morbidity of radiotherapy, thus improving quality of life in cancer survivors.

## Conclusion

Image-guided radiotherapy based on helical Tomotherapy may provide significant reduction of radiation dose to the contralateral parotid gland for patients with head and neck cancers without any compromise of target coverage or excessive radiation to other normal structures. This innovative technique should be investigated to assess if it can improve patient quality of life following head and neck radiotherapy.

## Competing interests

The authors have no competing interest and have no source of funding.

## Authors’ contributions

NPN, BS, and AC collected the data. PV performed the statistical analysis. All authors participated in the study design, data interpretation, and writing of the draft. All authors read and approve the manuscript.

## Pre-publication history

The pre-publication history for this paper can be accessed here:

http://www.biomedcentral.com/1471-2407/12/175/prepub
